# Welcoming articles on genotype-dependent clinical features and diagnostics

**DOI:** 10.1007/s10048-021-00638-5

**Published:** 2021-04-01

**Authors:** Georg Auburger, Manuel B. Graeber, Louis J. Ptáček

**Affiliations:** 1 Experimental Neurology, Medical Faculty, Goethe University, 60590 Frankfurt, Germany; 2 Brain and Mind Centre, University of Sydney, 94 Mallet St, Camperdown, NSW 2050 Australia; 3 Department of Neurology, University of California, San Francisco, USA

The rapid expansion of molecular genetics was catalyzed in 1949 by the first identification of an “allelic change in a single gene”, the E6V substitution in hemoglobin that is responsible for sickle cell anemia [1]. It was a surprise for many researchers then, that this disorder which is characterized by many crises of pain due to vaso-occlusion in fingers, chest, spleen or penis, by aplastic or hemolytic crises, and by stroke episodes, should be due to a constitutively present mutation. Decades later, neurogenetic research documented many variants of epilepsy and episodic ataxia, dyskinesia and paralysis to be caused by constitutive mutations in ion channels or other synaptic machinery [2]. It was also surprising that the hemoglobin aggregation underlying the sickle cell trait appears only in venous blood and readily disappears in reoxygenated arterial blood [3]. Again, neurogenetic research decades later showed how mutation-triggered protein aggregates that underlie age-associated neurodegeneration and dementia appear preferentially in neurons when glutamate/calcium-dependent environmental conditions are propitious [4]. These are examples that illustrate how the identification of causal mutations made it possible to experimentally unravel disease mechanisms, and thus to explain the associated clinical phenotypes, overall improving patient diagnostics and preventive treatment. Since its foundation in 1997, the journal *Neurogenetics* is committed to facilitating such research into rare genetic disorders of the nervous system.

In the past, exome sequencing of single cases has often left causality in doubt until in vitro experiments added the necessary evidence or animal models were generated that recapitulated the phenotype. Similarly, association studies of complex multifactorial conditions often led to spurious significance, which could not be confirmed. It is one of our task as editors to help weed out such inconclusive results.

In recent years, a sufficient number of cases even of rare clinical entities have become known to allow confident conclusions about their respective phenotype spectrum, their prodromal features and common treatment complications; enough mutations are known to understand the aberrant activity or interactions of each disease protein; enough disease genes are known to evaluate the pathogenetic pathways of a syndrome; enough expression profiles from peripheral tissues are documented to establish diagnostic criteria; therefore, we encourage authors to not only report some novel findings in a few of their patients, but also to embark on their comparison and comprehensive analysis. It is also desirable to go beyond DNA studies, either establishing additional diagnostic signatures, or analyzing pathogenesis and disease rescue in model systems.

With these goals in mind, we welcome the insights gained from recent *Neurogenetics* articles on expanded phenotypes in spastic paraplegia [5], on prodromal cognitive symptoms in SCA48 [6], on neuroimaging correlates of MYORG mutations [7] and of C1R mutations [8], on the oxidative stress triggered by PTCD3 mutations [9], on cell morphology analyses in ARSACS patient cells [10], on the interaction with transcription factors of CtBP1 [11], and on expression profiles modulated by the NDRG family [12].

We strongly invite submission of more such articles. Many thrilling developments continue to happen in our field, and we are eager to further expand the scope of *Neurogenetics* especially in the clinical arena in the coming years.
